# Identification of microRNAs in *Macaca fascicularis* (Cynomolgus Monkey) by Homology Search and Experimental Validation by Small RNA-Seq and RT-qPCR Using Kidney Cortex Tissues

**DOI:** 10.1371/journal.pone.0142708

**Published:** 2015-11-12

**Authors:** Yaligara Veeranagouda, Pierrick Rival, Catherine Prades, Claire Mariet, Jean-François Léonard, Jean-Charles Gautier, Xiaobing Zhou, Jufeng Wang, Bo Li, Marie-Laure Ozoux, Eric Boitier

**Affiliations:** 1 Sanofi R&D, Disposition Safety and animal Research, Vitry-sur-Seine, France; 2 Sanofi R&D, Global Biotherapeutics, Vitry-sur-Seine, France; 3 National Center for Safety Evaluation of Drugs (NCSED), National Institutes for Food and Drug Control, Beijing, China; Huazhong University of Science and Technology, CHINA

## Abstract

MicroRNAs (miRNAs) present in tissues and biofluids are emerging as sensitive and specific safety biomarkers. MiRNAs have not been thoroughly described in *M*. *fascicularis*, an animal model used in pharmaceutical industry especially in drug safety evaluation. Here we investigated the miRNAs in *M*. *fascicularis*. For *Macaca mulatta*, a closely related species of *M*. *fascicularis*, 619 stem-loop precursor miRNAs (pre-miRNAs) and 914 mature miRNAs are available in miRBase version 21. Using *M*. *mulatta* miRNAs as a reference list and homology search tools, we identified 604 pre-miRNAs and 913 mature miRNAs in the genome of *M*. *fascicularis*. In order to validate the miRNAs identified by homology search we attempted to sequence miRNAs expressed in kidney cortex from *M*. *fascicularis*. MiRNAs expressed in kidney cortex may indeed be released in urine upon kidney cortex damage and be potentially used to monitor drug induced kidney injury. Hence small RNA sequencing libraries were prepared using kidney cortex tissues obtained from three naive *M*. *fascicularis* and sequenced. Analysis of sequencing data indicated that 432 out of 913 mature miRNAs were expressed in kidney cortex tissues. Assigning these 432 miRNAs to pre-miRNAs revealed that 273 were expressed from both the -5p and -3p arms of 150 pre-miRNAs and 159 miRNAs expressed from either the -5p or -3p arm of 176 pre-miRNAs. Mapping sequencing reads to pre-miRNAs also facilitated the detection of twenty-two new miRNAs. To substantiate miRNAs identified by small RNA sequencing, 313 miRNAs were examined by RT-qPCR. Expression of 262 miRNAs in kidney cortex tissues ware confirmed by TaqMan microRNA RT-qPCR assays. Analysis of kidney cortex miRNA targeted genes suggested that they play important role in kidney development and function. Data presented in this study may serve as a valuable resource to assess the renal safety biomarker potential of miRNAs in Cynomolgus monkeys.

## Introduction

MicroRNAs (miRNAs) are a class of small (18–24 nucleotide long) RNAs that are involved in regulation of gene expression by targeting messenger RNAs (mRNA). Unlike other regulators, miRNAs exert highly complex combinatorial gene regulations by targeting hundreds of mRNA transcripts [1,2]. Among all miRNAs identified so far, those exhibiting high expression levels have been shown to be conserved across mammalian species [3]. Extensive research in the past decade indicates the involvement of miRNAs in various biological processes such cell development, proliferation, cell differentiation and apoptosis [1]. Dysregulation of miRNAs has been reported in many human diseases such as cancer, cardiovascular disease and autoimmune disorders [4,2]. Since miRNAs can be efficiently inhibited by modified/synthetic antisense oligonucleotides, there is great interest in developing anti-miRNA therapies for several diseases. For example, anti-miRNA therapies for treatment of liver cancer and hepatitis C infections are in clinical Phase I and Phase II respectively [5]. In addition to tissues/cells, miRNAs were also detected in various biofluids such as serum/plasma, urine, saliva, cerebrospinal fluid and amniotic fluid [6,7]. Usually miRNAs present in biofluids are packed in exosomes or associated to proteins or lipoproteins and hence protected from enzymatic degradation. Because of their stability and specificity, several studies demonstrated the utility of circulating miRNAs as diagnostic and prognostic biomarkers in disease such as cancer, cardiac disease and autoimmune disease [8,9 and 10]. More recently, microRNAs have been investigated as potential safety biomarkers [11]. Thus miRNAs received much attention not only as global intracellular and intercellular regulators but also as therapeutic targets and disease or safety biomarkers [11]. Hence there is great interest in miRNA identification and profiling.

*Macaca fascicularis* (also known by different names: Cynomolgus monkey, long-tailed macaque, crab-eating macaque) belongs to Cercopithecinae subfamily of the old world monkeys and native to Southeast Asian countries such as Indonesia and Philippines [12]. It emerged as an alternative model animal when *Macaca mulatta* (Rhesus macaque) was banned from India. As a non-human primate species, *M*. *fascicularis* has been used in pharmaceutical research, especially for the safety evaluation of biopharmaceuticals. Use of *M*. *fascicularis* in preclinical drug safety evaluation promoted the sequencing of its genome. As a result, first *M*. *fascicularis* of Mauritius origin was sequenced by a shotgun sequencing approach [13]. Comparative genomic analysis indicated that the genome of *M*. *fascicularis* exhibited 99.2% and 92.8% identity to *M*. *mulatta* and *H*. *sapiens*, respectively. Comparison of 10,919 mRNAs between *M*. *fascicularis* and humans indicated higher sequence conservation in the coding DNA sequences and higher divergence in 5′ and 3′ untranslated regions (UTRs) [13]. This finding supported an earlier hypothesis of King and Wilson who proposed that organismal differences between human and chimpanzee resulted from changes in gene expression rather than changes (mutations) in protein coding sequences [14]. Since UTRs are involved in epigenetic imprints and harbor binding sites for transcription factors and miRNAs, they contribute to phenotypic and physiological differences and hence play critical role in evolution. In fact, a recent study involving humans and non-human primates (chimpanzee and Rhesus macaque) showed that miRNAs influence large proportions of genes especially by targeting transcription factor genes [15]. Although whole-genome assemblies are available for more than nine strains of *M*. *fascicularis*, relatively little is known about miRNAs in this species [13,16–18]. So far less than hundred miRNAs were proposed for *M*. *fascicularis* and many of them are not supported by systematic experimental data [19,20]. Studying miRNAs in *M*. *fascicularis* would not only help to understand evolutionary adaptations and disease models, but would also provide an opportunity for biomarker development, especially to monitor drug-induced toxicity. In view of drug development still there is a need for sensitive and specific nephrotoxicity biomarkers which can reliably indicate kidney injury in experimental animals and humans. Recently miRNAs have been identified as potential biomarkers of nephrotoxicity in rat [21,22]. Exploring urinary miRNAs as potential biomarkers of nephrotoxicity in *M*. *fascicularis* may have translational applications in humans. Thorough evaluation of kidney and urinary miRNAs in *M*. *fascicularis* may provide a basis for the development of novel nephrotoxicity biomarkers which could be used in safety evaluation of biopharmaceuticals and new drugs.

In this study, we investigated the miRNAs in *M*. *fascicularis* genome by homology search and attempted to validate miRNAs by small RNA sequencing and RT-qPCR using kidney cortex tissues. We focused on miRNAs in kidney cortex as miRNAs expressed in this tissue could be released into urine upon tissue damage and thus be used as potential non-invasive biomarkers of drug induced kidney injury in this species.

## Results

### Identification of miRNAs in *M*. *fascicularis* genome by homology search

**Fig 1:**
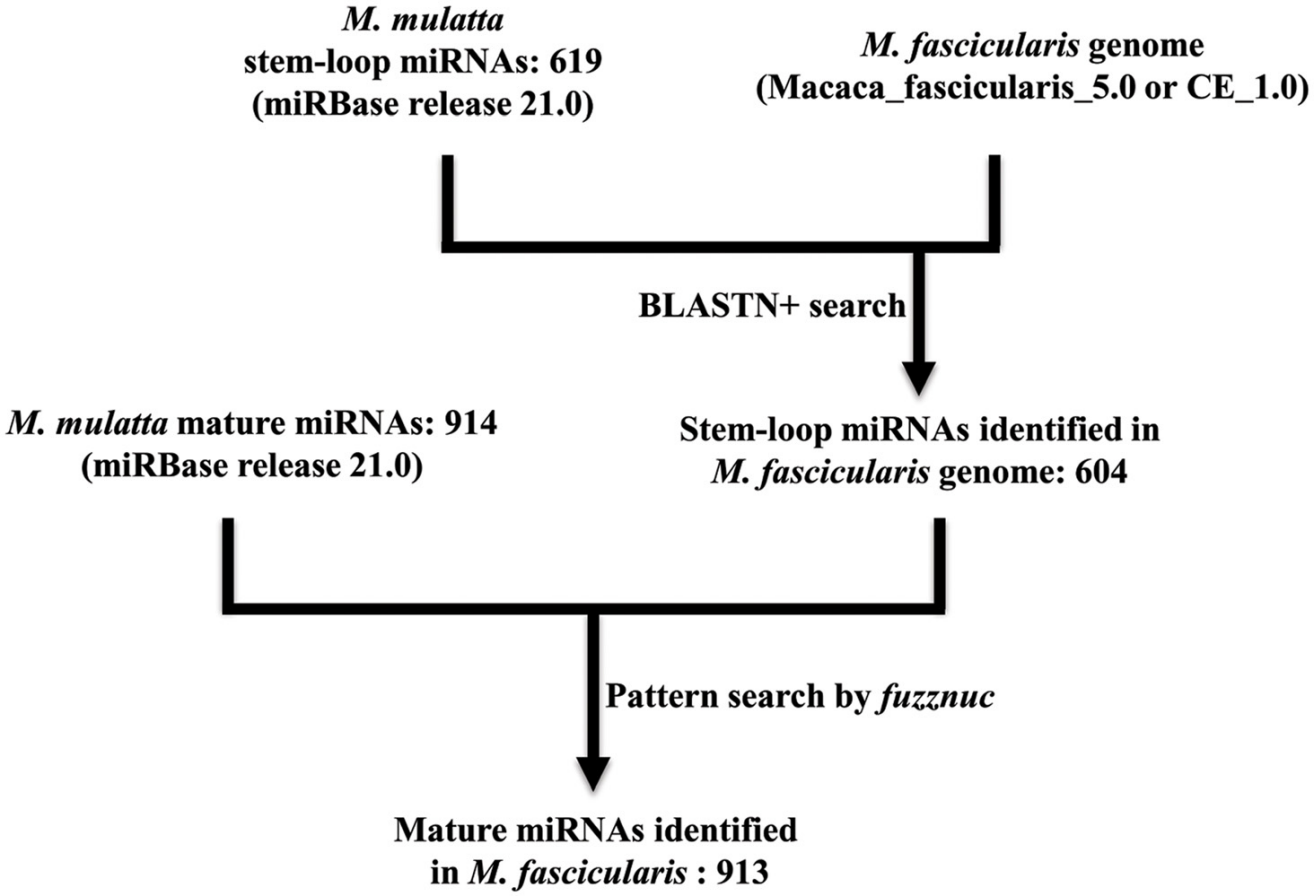
Workflow used for the identification of *M*. *fascicularis* miRNA genes.

When compared to the human genome, *M*. *fascicularis* shares a higher degree of sequence homology with *M*. *mulatta* (99.2% versus 92.8%). Hence we used stem-loop pre-miRNA and mature miRNAs from *M*. *mulatta* as a reference list to search homolog candidates in *M*. *fascicularis* genome. Previously Yue et al. identified 454 miRNAs in *M*. *mulatta* genome using human miRNAs as a reference [23]. In its recent release, miRBase (version 21) contains 619 stem-loop pre-miRNAs and 914 mature miRNAs from *M*. *mulatta* [24]. The workflow used for the identification of *M*. *fascicularis* miRNAs is shown in Fig 1. For stem-loop pre-miRNA identification, Macaca_fascicularis_5.0 (Washington University) and CE_1.0 (Beijing Genomics Institute) genomes were used as first and second choice respectively as the former genome was better annotated than the latter. To avoid too many false positive hits with mature miRNAs (due to short length), *M*. *mulatta* stem-loop sequences were used as queries for the BLASTN search against *M*. *fascicularis* genome. The criteria used for searching stem-loop pre-miRNAs in *M*. *fascicularis* genome were the following ones: 1) should match (≥90%) to full length stem-loop miRNA of *M*. *mulatta*, 2) no multiple genomic locations with same number of mismatches, 3) no same genomic location for same family members, and 4) manual curation of BLASTN results if the first 3 criteria failed. With these criteria, 604 stem-loop pre-miRNA genes were identified in *M*. *fascicularis* genome. Out of these 604 genes, 528 had 0 mismatch, 58 had one mismatch and the remaining 18 had 2–5 mismatches with respect to *M*. *mulatta* stem-loop miRNA genes. Details of identified *M*. *fascicularis* stem-loop pre-miRNA gene sequences, proposed names, chromosomal location and strand information are provided in S1 Table.

In order to infer *M*. *fascicularis* mature miRNAs, *M*. *mulatta* mature miRNA sequences were compared against corresponding *M*. *fascicularis* stem-loop pre-miRNA genes using *fuzznuc* algorithm. With this approach, out of the 914 mature *M*. *mulatta* miRNAs, 913 were identified in *M*. *fascicularis*. When compared to *M*. *mulatta* mature miRNA sequences, majority of *M*. *fascicularis* miRNAs (881) had identical sequences (0 mismatch). The rRemaining 28, 2 and 2 *M*. *fascicularis* miRNAs exhibited 1, 2 and 3 mismatches, respectively. The identified miRNA sequences, proposed names, and length of miRNAs are shown in S2 Table. Thus, using *M*. *mulatta* miRNA reference sequences, we identified 604 stem-loop and 913 mature miRNAs in *M*. *fascicularis* genome.

Previously, Yang et al. [20] identified 86 miRNAs in *M*. *fascicularis* using transcriptome data from five different tissues (brain, ileum, kidney, liver, testes and white adipose tissue) [20]. We compared the lists of miRNAs identified in this study with the previously predicted 86 miRNAs using BLASTN+. Only 39 miRNAs were mapped to either stem-loop or mature miRNAs and the remaining 47 were not present in the current miRNA list (S3 Table).

### Comparison of *M*. *fascicularis* miRNAs against human miRNAs

In order to determine the identity between miRNAs from *M*. *fascicularis* and *H*. *sapiens*, the *M*. *fascicularis* mature miRNAs were aligned to their human counterparts (i.e. same miRBase name/family) to highlight the identity and potential overlapping between these two species. Interestingly 763 of the 913 *M*. *fascicularis* mature miRNAs were found in human miRNAs: 417 had perfect match across the full length, 150 had 0 mismatch across >15 internal nucleotides, and except mfa-miR-607 which had 10 mismatches, the remaining 195 had 1 to 5 mismatches with same length (S4 Table). Thirty-five *M*. *fascicularis* mature miRNAs could be mapped to human stem-loop pre-miRNAs with less than 2 mismatches (S5 Table). The remaining 115 mature miRNA sequences, corresponding to 67 *M*. *fascicularis* stem-loop miRNA sequences were screened against the human genome (GRCh38.p2) to determine if human homologues could be observed (S6 Table). At least 46 of them were present in the human genome. These results indicate that the majority of *M*. *fascicularis* miRNAs are conserved in the human genome.

### Confirmation of *M*. *fascicularis* miRNAs by small RNA-sequencing

Next, we attempted to experimentally validate the newly-discovered *M*. *fascicularis* miRNAs by small RNA sequencing. For this purpose, small RNA sequencing libraries were prepared from three naive *M*. *fascicularis* kidney cortex tissues and sequenced as described in Methods section. On average, we obtained 36 million reads for each kidney cortex tissue sample (Table 1). RNA sequencing reads with a length below 18 nucleotides were removed and remaining reads were mapped to the 913 *M*. *fascicularis* miRNAs. We considered miRNAs as genuine only when they had a minimum of 15 combined sequencing reads from the three kidney cortex tissues analyzed. With this criterion we identified 432 and 447 miRNAs with 0 and 1 mismatch, respectively; 432 miRNAs were present in both groups and 15 were unique to 1 mismatch list (Table 1). Although mapping with 1 mismatch increased the total number of mapped reads (from 12.7 to 14.2 million), it only yielded 15 additional unique miRNAs; 14 of them had 15–32 reads. In fact, all 15 miRNAs identified with 1 mismatch were also present in the 0 mismatch list but had 7–14 reads; hence they did not pass the set criteria. From this result it is clear that allowing 1 mismatch only contributed to the increase in sequencing reads to the existing miRNAs rather than discovery of new miRNAs. Thus in subsequent studies, we retained miRNAs with 0 mismatch (432 miRNAs—S7 Table).

**Table 1:** Mapping RNA sequencing reads to *M*. *fascicularis* miRNAs.

	Kidney cortex tissues			Mean	Sum	miRNAs identified
	K1	K2	K3			
Sequencing reads	38 777 154	36 789 725	32 525 071	36 030 650	108 091 950	-
Reads after filtering <18 nucleotides	12 024 675	14 912 774	15 272 440	14 069 963	42 209 889	-
Mapping to miRNAs with 0 mismatch	4 230 817	4 625 742	3 911 065	4 255 875	12 767 624	432
Mapping to miRNAs with 1 mismatch	4 760 282	5 262 441	4 406 932	4 809 885	14 429 655	447

### Assigning 432 miRNAs to pre-miRNAs

**Fig 2:**
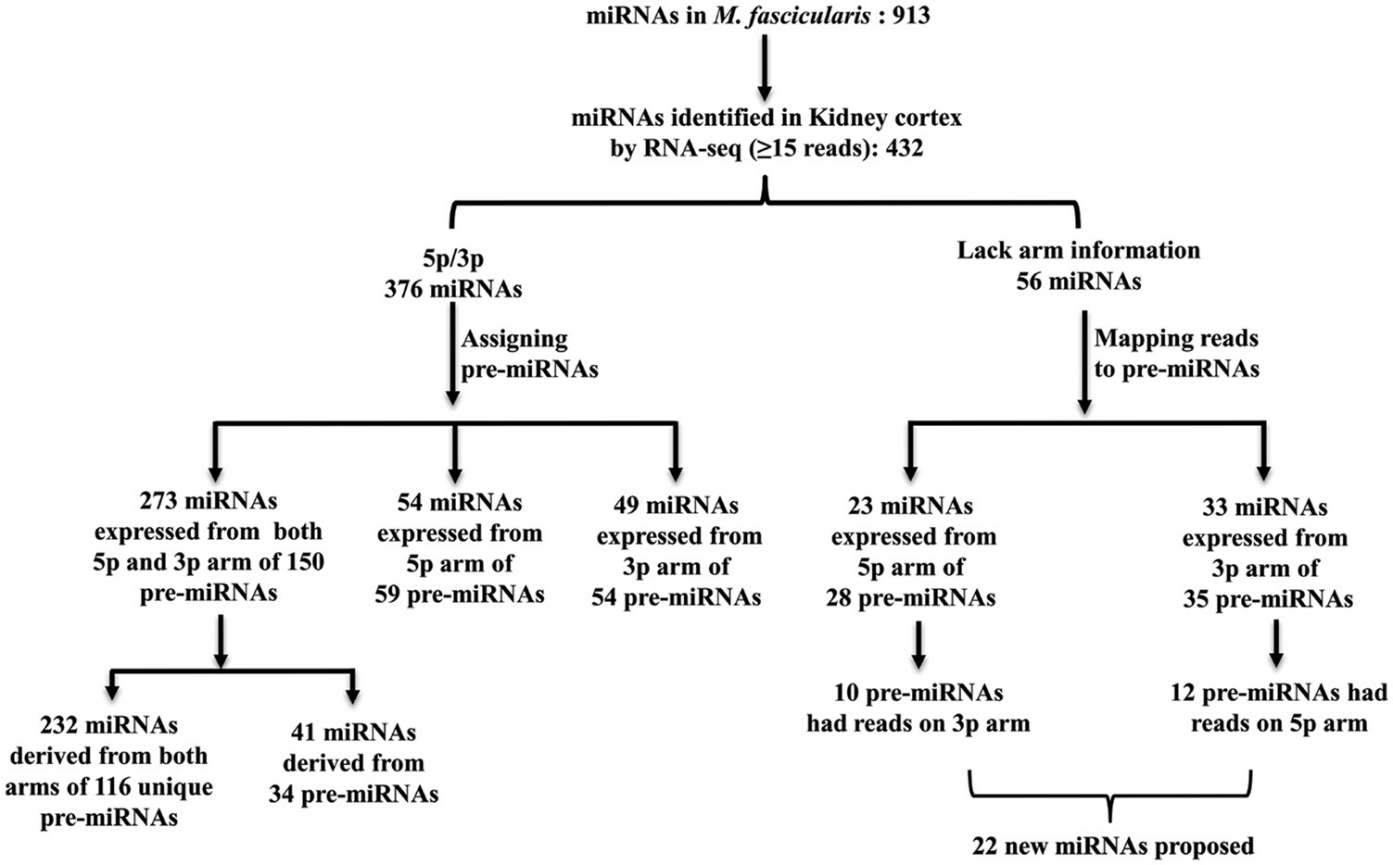
Mapping 432 expressed miRNAs to respective pre-miRNAs.

Generally, miRNAs are represented with suffix -5p or -3p only when a stem-loop pre-miRNA produces mature sequences from both -5p and -3p arms. On the other hand, if a pre-miRNA produces only one mature microRNA, arm is not usually specified [24]. In our *M*. *fascicularis* list resulting from the initial analysis, 687 of 913 miRNAs were indicated with a -5p or -3p suffix. The remaining 226 miRNAs lacked the arm information. In order to understand whether miRNAs identified in kidney cortex by RNA sequencing resulted from single or both arms of a given precursor, we sorted the 432 mature miRNAs based on stem-loop pre-miRNA information. 376 out of the 432 miRNAs presented either a -5p or -3p in their nomenclature and the remaining 56 miRNAs lacked the arm information (Fig 2). Then we assigned the 376 miRNAs to respective pre-miRNAs using S1 Table and examined for the presence/absence of mature sequence/reads on each arm. As shown in Fig 2, 273 out of 376 miRNAs were expressed from both arms of 150 pre-miRNAs: 232 were derived from both arms of 116 unique pre-miRNAs (-5p or -3p miRNAs derived from unique/ single pre-miRNA) and 41 miRNAs derived from both arms of 34 pre-miRNAs (-5p or -3p mature miRNAs derived from more than one pre-miRNA). In addition, 54 miRNAs were expressed from only the -5p arm of 59 pre-miRNAs and 49 miRNAs resulted from only the -3p arm of 54 pre-miRNAs. Hence these 113 mature miRNAs (54 from -5p arm and 49 from -3p arm) derived from only one arm of pre-miRNAs and the corresponding mature miRNA counterparts were not expressed in the present dataset.

For the remaining 56 miRNAs which lacked arm information, instead of assigning them to pre-miRNAs (S1 Table), FASTQ files were mapped against pre-miRNA sequences. This not only helped to assign arm information, but also allowed to visualize reads on both arms of pre-miRNAs. As shown in Fig 2, 23 of 56 miRNAs were expressed from the -5p arm of 28 pre-miRNAs. Interestingly, 10 of the 28 pre-miRNAs also had reads (>15) on the -3p arm and these miRNAs were not described in *M*. *mulatta*. Thirty-three of 56 miRNAs were expressed from the -3p arm of 35 pre-miRNAs. Here also 12 of the 35 pre-miRNAs had reads (>15) on the -5p arm (which have also not been described before in *M*. *mulatta*). We retrieved conserved miRNA sequences from 22 newly identified mature miRNAs (10 miRNAs from -5p arm and 12 miRNA from -3p arm) and searched in miRBase (S8 Table). Interestingly all 22 miRNAs were present in human and other species with little variation in nucleotide sequence (data not shown).

From the above results it is clear that many *M*. *fascicularis* miRNAs identified by homology search were present / expressed in kidney cortex tissue. Altogether 432 mature miRNAs expressed in kidney cortex were derived from 326 pre-miRNAs and many of them were expressed from both arms of pre-miRNA (S8 Table). In addition, mapping sequencing reads to the pre-miRNAs allowed us to propose 22 new miRNAs which were not previously described in *M*. *mulatta*.

### RT-qPCR validation of *M*. *fascicularis* miRNAs

In order to confirm the miRNAs identified by RNA sequencing, we performed RT-qPCR analysis on some of the miRNAs. TaqMan Low Density Array (TLDA) cards have 754 RT-qPCR assays specific to human miRNAs (TLDA-A and B cards). Comparison of the 754 human mature miRNA sequences to the 913 *M*. *fascicularis* miRNA sequences indicated that at least 294 miRNAs had exact match and 19 had 1 mismatch (total 313 miRNAs). Hence we used TLDA cards to examine 313 *M*. *fascicularis* miRNAs. Total RNA prepared from 3 kidney cortex tissues was subjected to RT-qPCR using human TLDA A and B cards as described in Methods section. The Ct (cycle threshold) values obtained from the 3 tissues were averaged and miRNAs with Ct ≤ 32 were considered as expressed. Using this criterion, 262 of 313 *M*. *fascicularis* miRNAs were detected by RT-qPCR (S9 Table).

**Fig 3:**
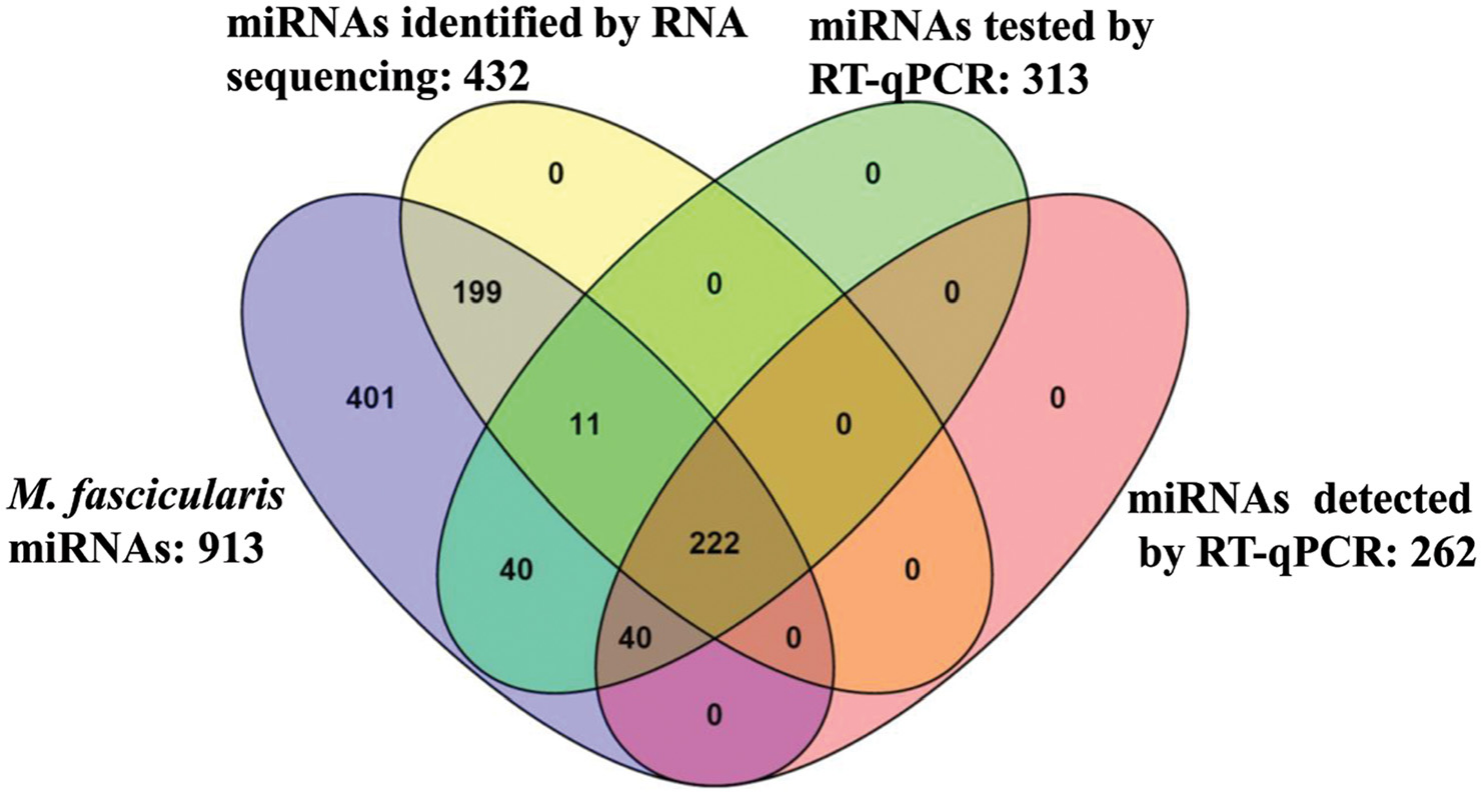
Comparison of *M*. *fascicularis* miRNAs identified by RNA sequencing and RT-qPCR.

Next we compared miRNAs identified by sequencing and qRT-PCR. As shown in Fig 3, 432 and 262 miRNAs were detected in *M*. *fascicularis* kidney cortex tissues by RNA sequencing and RT-qPCR, respectively; 222 of 913 miRNAs were validated by both RNA sequencing and RT-qPCR (S10 Table). Interestingly 11 miRNAs identified by RNA sequencing were not detected by RT-qPCR. Also 40 miRNAs identified by RT-qPCR were not detected by RNA sequencing. The observed discrepancy may be due to the differences in sample processing and sensitivity of each platform.

### Expression analysis and target prediction

**Fig 4:**
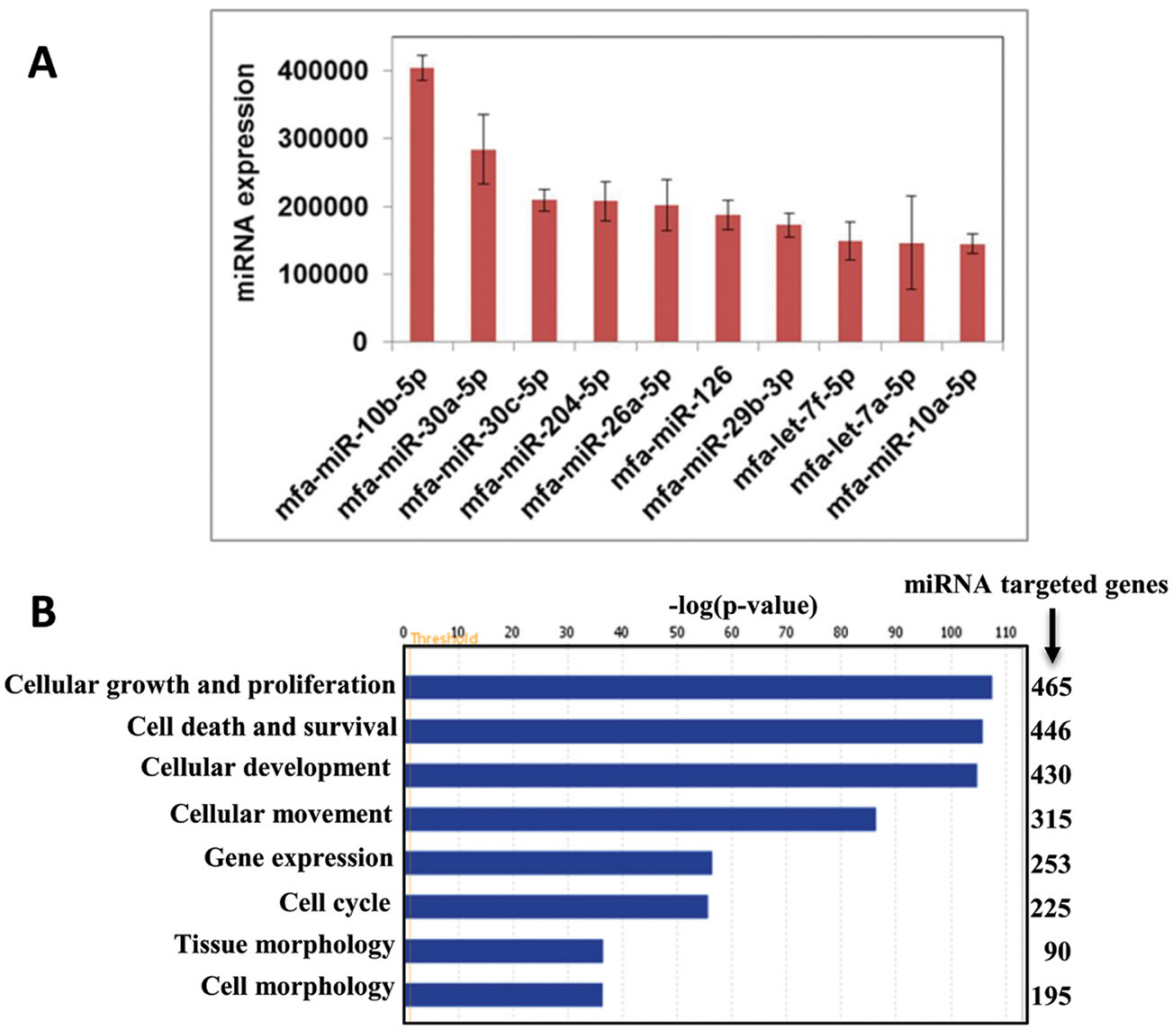
A. Top 10 highly expressed miRNAs in kidney cortex from *M*. *fascicularis*. B. Functions of kidney cortex miRNAs targeted genes.

For the expression analysis, read counts obtained from the three kidney cortex tissues were normalized with edgeR package and examined for kidney specific miRNAs (S11 Table). Several of the 432 miRNAs had been previously reported to be kidney-specific miRNAs: miR-10b-5p, miR-30c-5p, miR-204-5p, miR-10a-5p, miR-30d-5p, miR-200a-3p, miR-196a-5p, miR-196b-5p and miR-146a-5p [25]. Many of those miRNAs were among the top 10 highly expressed miRNAs in kidney cortex samples (Fig 4A). Enriched expression of a cluster of miRNAs such as miR-192, miR-194, miR-204 and miR-215 in human kidney tissue was previously confirmed by Northern blot analysis [26]. Expression of miR-23, miR-24, miR-26a, miR-10a and miR-30c in glomerulus and renal tubules has also been previously reported in mice [27]. Also involvement of miR-30 family in kidney development is documented [28].

Next, to understand the functional relevance of miRNAs expressed in kidney cortex, we attempted to link the miRNAs to target genes. Since most of the target prediction tools are designed for human miRNAs, expressed *M*. *fascicularis* kidney cortex miRNAs which had human orthologs (392 miRNAs) were used (S4 Table) in further analysis. Using Ingenuity Pathway Analysis solftware, only experimentally observed miRNA-target genes interactions in Human were considered. With these setting 392 miRNAs yielded 1084 target genes. Kidney cortex miRNA targeted genes associated with molecular and cell functions are shown in Fig 4B. Especially, miRNA targeted genes with lowest p-value are involved in basic cellular processes such as cell growth and proliferation, cellular development, tissue morphology and cell death. Overall results of target analysis indicate that miRNAs expressed in kidney cortex are involved in regulation of various key cellular processes in kidney cortex cells from *M*. *fascicularis*.

## Discussion

In this study we systematically investigated the miRNAs in *M*. *fascicularis*, an animal model with high translational potential to human. By comparing metazoan miRNAs sequence to expressed sequence tags (EST) of *M*. *fascicularis*, Yang et al. (2012) identified eight miRNAs in *M*. *fascicularis* [19]. With the exception of one (rno-miR-3591), all miRNAs described in this previous study are present in our current list of 913 *M*. *fascicularis* mature miRNAs. More recently, Yang et al. (2014) identified 86 putative miRNAs in *M*. *fascicularis* by transcriptome analysis [20]. Interestingly only 39 out of 86 miRNAs were present in our list of 913 *M*. *fascicularis* mature miRNAs and 47 were absent (S3 Table). This discrepancy may be mainly due to the difference in workflows used in each study. Indeed, Yang et al. (2014) used all existing metazoan miRNA sequences to map their transcriptome data against, whereas we followed a more focused and specific approach using *M*. *mulatta* stem-loop pre-miRNA sequences against *M*. *fascicularis* genome.

Results presented in this investigation indicate that *M*. *mulatta* miRNAs are found to be highly conserved in *M*. *fascicularis* as the vast majority of *M*. *mulatta* miRNA sequences are identical to miRNA sequences identified in *M*. *fascicularis* genome (881 out of 913 miRNAs). This result is consistent with the fact that both macaque species exhibit high degree of genome identity (99.2% identical) [13]. Also the majority of *M*. *fascicularis* miRNAs are conserved in *Homo sapiens*. A recent miRNA expression study in human and non-human primates showed positive correlations between miRNA sequence conservation and expression levels. Highly conserved miRNAs exhibited higher levels of expression in human, chimpanzee and rhesus macaque [15]. Conversely negative correlation was observed between miRNA hairpin SNP number and miRNA expression. Similar results were also observed in humans: less expressed miRNA showed weaker selection and hence had less constraint evolution than highly expressed miRNAs [29]. High conservation of miRNAs among *M*. *fascicularis*, *M*. *mulatta* and human may reflect similar regulatory networks across these three primate species.

Recently, miRBase (version 21) set several criteria to distinguish authentic miRNAs from false annotations. One of the criteria for high confidence miRNA is that pre-miRNA should have at least 10 reads corresponding to each of the two possible mature miRNA sequences [24]. Sequencing of kidney cortex miRNAs at high depth (total 12.7 million miRNA reads/3 animals) not only facilitated the sequencing of a large number of miRNAs but also aided the detection of both -5p and -3p mature sequences of pre-miRNAs. Using stringent mapping criteria, 432 miRNAs were detected in kidney cortex tissue; 273 out of 432 miRNAs derived from both -5p and -3p arms of 150 pre-miRNAs and in most cases only one arm showed dominant expression. Thus our results confirm the expression of both -5p and -3p arm of 150 pre-miRNAs (S8 Table). Recent progress in miRNA research indicated that mature miRNAs produced from both arms (-5p and -3p) of pre-miRNAs are biologically active and expression of a dominant mature sequence varies with developmental stages, tissue type and even with species [30].

Tissue-specific miRNAs and their target genes play vital role in organ or tissue differentiation, development and function. MiRNAs expressed in kidney cortex of *M*. *fascicularis* described in this study are in good agreement with previously reported kidney specific miRNAs [25]. Many of those kidney specific miRNAs were expressed at very high level and their target genes were involved in various basic cellular functions indicating that they play vital role in kidney. Interestingly, in our preliminary investigation on urinary miRNAs in *M*. *fascicularis* conducted in the context of the Health and Environmental Sciences Institute (HESI) Biomarkers of Nephrotoxicity Committee we observed 9 out of top 10 kidney cortex miRNAs (Fig 4A) in urine samples of *M*. *fascicularis* and they were also among the top 10 highly represented urinary miRNAs (data not shown). Similar miRNAs were also reported for human urine: hsa-miR-30a-5p, hsa-miR-10b-5p, hsa-miR-10a-5p, hsa-miR-26a-5p and hsa-miR-30c-5p are among the 10 highly represented miRNAs in exosomal preparation of human urine [31]. Thus kidney (cortex, medulla and papilla) miRNA profiles may help to understand miRNAs present in urine.

In conclusion we identified 913 miRNAs in *M*. *fascicularis* and experimentally validated 432 miRNAs in kidney cortex tissues by RNA-sequencing. In addition expression of 222 miRNAs was confirmed by both RNA sequencing and RT-qPCR. The data presented in this investigation will serve as valuable resource especially for exploring biomarker potential of miRNAs in *M*. *fascicularis*. Urinary miRNAs indeed hold great promise as kidney safety biomarkers [32]. Following this line of thought, we are currently exploring biomarker potential of urinary miRNAs in *M*. *fascicularis* treated with various nephrotoxicants.

## Materials and Methods

### Animals and Ethics Statement

All animal experiment protocols were reviewed and approved by the Institutional Animal Care and Use Committee (IACUC) of National Center for Safety Evaluation of Drugs (NCSED). Cynomolgus monkeys (ages 2–3 years old) were maintained in stainless steel cages (L×W×H: 800×700×750 mm) under condition of 16–26°C, 40–70% relative humidity, a 12-h light-dark cycle and a room air exchange of 8–10 times per hour. Each monkey was provided with 300 g of standard monkey keeping diet and fruits per day, and sterilized water was offered ad libitum. The monkey was individually housed with toys (such as mirror) and monitored daily by the animal care staff. At the end of this experiment, monkeys were exsanguinated following the deep anesthesia by IV injection of pentobarbital sodium, and kidney cortex samples were collected, transferred to cryotubes containing RNALater reagent and stored at -80°C.

### RNA extraction, qualitative and quantitative analysis

Frozen kidney cortex samples (-80°C) were thawed on ice and used for total RNA extraction. Briefly, 25–30 mg of kidney cortex tissue were suspended in 700 μl QIAzol Lysis Reagent (Qiagen) and homogenized using TissueLyser for 90 seconds. Total RNA was extracted from the tissue homogenate using miRNeasy mini kit (Qiagen). The quality of RNA was checked using Agilent RNA 6000 Nano kit and 2100 Bioanalyzer (Agilent). All RNA samples had a RNA integrity number between 7 to 8. The RNA quantity was determined using RediPlate^™^ 96 RiboGreen^®^ RNA Quantitation kit (Thermo Fisher).

### Small RNA enrichment and sequencing library preparation

Five micrograms of total RNA were used for small RNA enrichment. Small RNA enrichment was performed using Total RNA-Seq kit v2 (Life Technologies) as per manufacturer’s recommendations. The sequencing library was prepared from 12 ng (quantified using High Sensitivity DNA analysis kit and 2100 Bioanalyzer) of enriched small RNAs using Total RNA-Seq kit v2 with custom barcode primers.

### Sequencing template preparation, Ion PI Chip loading and sequencing

Seventy μl (75 pM) of the sequencing library were used for template preparation. Sequencing templates were prepared using ION PI IC 200 kit (Life Technologies) and Ion Chef System (Life Technologies). One Ion PI Chip kit v2 BC (Life Technologies) was used for each sequencing library. Sequencing templates loaded on Ion PI Chip were sequenced using ION PI IC 200 kit and Ion Proton System (Ion Proton Sequencer and Ion Proton Torrent Server, Life Technologies), according to manufacturer’s protocol. The Ion Proton Torrent Server trims (removes sequencing key, barcode and adaptor sequences, lower-quality -3p ends with low quality scores) and filters (reads with < 8 nucleotides, adaptor dimers, reads lacking key, polyclonal reads, reads with low signal) the sequencing reads and generates FASTQ files.

### Analysis of sequencing data

FASTQ files were analyzed using Array studio V.8.0.3.67 (OmicSoft Corp). In the preprocessing step, reads with a length below 18 nucleotides and average quality score below 20 were removed from FASTQ files. Then FASTQ files were mapped against *M*. *fascicularis* miRNA reference sequences (generated in this study) and resulting BAM files were used for read quantitation. Generated miRNA reads were normalized by edgeR method.

### RT-qPCR using TaqMan MicroRNA Arrays

Complementary DNA was synthesized from kidney cortex total RNA (200 ng) using TaqMan MicroRNA Reverse Transcription kit (Life Technologies) and Megaplex^™^ TLDA A v2.1 and TLDA B v3.0 RT Primers. Resulting cDNA was used for qPCR. Quantitative real-time PCR was performed using TaqMan Universal Master Mix II (Life Technologies) and TaqMan TLDA Human MicroRNA A version 2.0 and TLDA Human MicroRNA B version 3.0 cards. Sample loaded TLDA cards were run on QuantStudio 12K Flex real-time PCR system (Life Technologies). Generated raw data files were analyzed using Expression Suite Software v1.0.3 (Life Technologies).

### miRNA target prediction

Only Human orthologs of *M*. *fascicularis* miRNAs expressed in kidney cortex were used for target prediction. A total of 392 miRNAs were used for target prediction using Ingenuity Pathway Analysis software version 23814503 (QIAGEN). Initial analysis yielded 17 000 predicted target messenger RNAs. Filters were applied to focus on a reduced but more relevant target gene set. The relationships column was refined with the selection of Ingenuity Expert, Expert Assist findings and miRecords, including experimentally observed and / or high or moderate prediction. With these settings a finalized list of 1084 target genes was obtained. Core analysis was carried out on this list using the following filters: direct and indirect relationships, microRNA-mRNA interactions including miRecords, TargetScan human and Tarbase. Only the human species was taken into account and the confidence level selected was “experimentally observed”. The chart corresponding to Molecular and Cell Function was customized to display biofunctions only.

### Bioinformatics analysis

For the homology search, BLASTN package was used [33]. *M*. *fascicularis* mature miRNAs were extracted with fuzznuc, a string-search algorithm from EMBOSS package by comparing each *M*. *mulatta* mature miRNA against each corresponding *M*. *fascicularis* predicted stem-loop [34]. Venn diagram was constructed using VENNY 2.0 [35].

## Supporting Information

S1 Table Identification of *M*. *fascicularis* stem-loop miRNAs by BLASTN searching of M. mulatta stem-loop miRNA reference sequences in *M*. *fascicularis* genome. (XLSX)

S2 Table Identification of *M*. *fascicularis* miRNAs by comparing *M*. *mulatta* miRNA reference sequence against *M*. *fascicularis* stem-loop miRNA sequences. (XLSX)

S3 Table Comparison of *M*. *fascicularis* miRNAs with previously reported 86 M. fascicularis miRNAs. (XLSX)

S4 Table Comparison of *M*. *fascicularis* miRNAs versus *H*. *sapiens* miRNAs. (XLSX)

S5 Table Comparison of *M*. *fascicularis* stem-loop miRNA sequences versus *H*. *sapiens* stem-loop miRNAs sequences. (XLSX)

S6 Table Comparison of *M*. *fascicularis* stem-loop miRNAs versus *H*. *sapiens* genome. (XLSX)

S7 Table Mapping reads from 3 kidney cortex samples (K1, K2 and K3) to *M*. *fascicularis* miRNAs with 0 mismatch. MiRNA reads were counted from 3 kidney cortex tissues. MiRNA having reads ≥ 15 were considered as genuine—With this criterion, a total of 432 mature miRNAs were identified.
(XLSX)

S8 Table Assigning reads to *M*. *fascicularis* stem-loop pre-miRNAs and mature miRNAs. (XLSX)

S9 Table Detection of miRNAs by RT-qPCR. TaqMan^®^ MicroRNA Arrays (Human Arrays A and B) were used for detection of miRNAs expressed in kidney cortex tissues. MiRNAs specific to *M*. *fascicularis* are shown in table. For each miRNA, average Ct is calculated from three kidney cortex samples (K1, K2 and K3). MiRNAs with Ct ≤32 were considered as expressed. With this criterion, a total of 262 miRNAs were considered as expressed.
(XLSX)

S10 Table Comparison of miRNAs between *M*. *fascicularis* miRNAs (913), miRNAs identified by small RNA sequencing (432), miRNAs tested by RT-qPCR (313) and miRNAs detected by RT-qPCR (262). (XLSX)

S11 Table EdgR normalized miRNAs values from three kidney cortex samples. *M*. *fascicularis* miRNAs ortholog to human miRNAs were used for target identification.
(XLSX)
