# Correction: Identification of microRNAs in *Macaca fascicularis* (Cynomolgus Monkey) by Homology Search and Experimental Validation by Small RNA-Seq and RT-qPCR Using Kidney Cortex Tissues

**DOI:** 10.1371/journal.pone.0164317

**Published:** 2016-10-03

**Authors:** Yaligara Veeranagouda, Pierrick Rival, Catherine Prades, Claire Mariet, Jean-François Léonard, Jean-Charles Gautier, Xiaobing Zhou, Jufeng Wang, Bo Li, Marie-Laure Ozoux, Eric Boitier

The following information is missing from the Data Availability section: FASTQ files have been deposited in GEO and can be accessed at http://www.ncbi.nlm.nih.gov/geo/query/acc.cgi?acc=GSE86836. Other relevant data is within the paper and Supporting Information files.
